# Supplementary material for: Health-related quality of life of the adult COVID-19 patients following one-month illness experience since diagnosis: Findings of a cross-sectional study in Bangladesh
Source: PLoS One. 2023 Feb 22;18(2):e0279032. doi: 10.1371/journal.pone.0279032 (PMC9946204; doi:10.1371/journal.pone.0279032)
Supplement: Attachment — Submitted filename: Plos one_reviewer comments.pdf [file pone.0279032.s002.pdf]

Reviewer Comments

The authors have done a great job by selecting a topic which is the need of the hour. There are very few studies conducted in this regard. Few of my comments are as follow:

1. The rationale of the study needs to be well elaborated.
2. Addition of studies using the same tool and specifically the used one will add more value.
3. The methodology needs to be technically more sound. The sample size calculation reference needs to be incorporated. How eligibility criteria was developed?
4. Results are quite confusing and need to be presented in a more understandable manner. The linear regression need to discussed in more detail with respect to different variables.
5. Discussion need to be rewritten.
6. Conclusion need to be rewritten with respect to study objectives.
7. The quality of writing in terms of English language is poor. The article needs major editing.
