# Supplementary material for: Health-related quality of life of the adult COVID-19 patients following one-month illness experience since diagnosis: Findings of a cross-sectional study in Bangladesh
Source: PLoS One. 2023 Feb 22;18(2):e0279032. doi: 10.1371/journal.pone.0279032 (PMC9946204; doi:10.1371/journal.pone.0279032)
Supplement: Attachment — Submitted filename: Response to Reviewers.docx [file pone.0279032.s004.docx]

**Responses to academic editor`s comments**

| **SL No** | **Comments** | **Responses** |
| --- | --- | --- |
| 1. | The rationale of the study and measures being used should be highlighted | Thank you for the very important comment. The ‘Rationale’ of the study and measures being used are highlighted. |
| 2. | The writing of Results and Discussions sections should be more clear and systematic | Thank you for the pertinent comment. The ‘Results’ and ‘Discussion’ section are rewritten to make it more clear and systematic. |
| 3. | Copyediting of English is a must. | Thank you for the vital comment. Copyediting of English is done. |
| 4. | A rebuttal letter that responds to each point raised by the academic editor and reviewer(s). You should upload this letter as a separate file labeled 'Response to Reviewers'. | Thank you for the crucial comment. A separate file labeled 'Response to Reviewers' is submitted. |
| 5. | A marked-up copy of your manuscript that highlights changes made to the original version. You should upload this as a separate file labeled 'Revised Manuscript with Track Changes'. | Thank you for the very valuable comment. A separate file labeled 'Revised Manuscript with Track Changes' is prepared and submitted. |
| 6. | An unmarked version of your revised paper without tracked changes. You should upload this as a separate file labeled 'Manuscript'. | Thank you for the crucial comment. A separate file labeled 'Manuscript' is prepared and submitted. |
| 7. | Please ensure that your manuscript meets PLOS ONE's style requirements, including those for file naming. | Thank you for the pertinent comment. The manuscript is formatted as per the requirement of PLOS ONE's style. |
| 8. | Upon re-submitting your revised manuscript, please upload your study’s minimal underlying data set as either Supporting Information files or to a stable, public repository and include the relevant URLs, DOIs, or accession numbers within your revised cover letter. | Thank you for the very valuable comment. The minimal underlying dataset of the study is shared with the submission under the heading of ‘Supporting Information file’. |
| 9. | Your ethics statement should only appear in the Methods section of your manuscript. If your ethics statement is written in any section besides the Methods, please delete it from any other section. | Thank you for the very important comment. Ethics statement is deleted from other sections besides the ‘Methods’ section. |
| 10. | Please include captions for your Supporting Information files at the end of your manuscript, and update any in-text citations to match accordingly. | Thank you for the major comment. The captions for the supporting information file S1 Data set (Deidentified Original Data set in SPSS) is included at the end of the manuscript. |

**Responses to reviewer-1 Comments**

| **SL No** | **Comments** | **Responses** |
| --- | --- | --- |
| 1. | Line 78: Please mention year along with the name of the month | Thank you for the crucial comment. Year is mentioned in the line 82. |
| 2. | The data presented in lines 79-80 may be updated | Thank you for the vital comment. Data is updated in the lines 83-84 and accordingly, the reference no. 4 is updated |
| 3. | What data did authors use to inform that in COVID-19 patients, the duration of 30 days after the RT-PCR is appropriate to assess the effect of disease on their HRQOL (as their HRQOL is stabilised / returned to baseline)? Is there a reference or any data to back up this determination? | Thank you for the vibrant comment. Health related quality of life is a multidimensional concept for examining the impact of health status on quality of life. CDC HRQOL-14 questionnaire is designed in a manner so that it can estimate the overall number of days during the previous 30 days when the respondent felt that either his or her physical or mental health was not good. The "number of days in the past 30 days" response format of the Healthy Days Measures makes them particularly well suited to respond to short-term changes in HRQOL. This instrument can calculate the HRQOL of 30 days, despite it has returned to baseline or stabilized. For this reason, it is appropriate to assess the condition of 30 days after diagnosis of COVID-19 as the disease has immediate impact on physical and mental condition of the patients.  Also the CDC HRQOL-14 is used for chronic health conditions such as arthritis, asthma, disability, depression, any emerging disease, communicable disease, emergency health event like disaster etc. and also it can be used in healthy population. The aggregated core data on ‘healthy days’ are responsive to the effect of a major disease or disaster. Details can be found in the article of Moriarty DG et al (Reference12 in the article). It is also mentioned in lines 535-536 under discussion section of the article. |
| 4. | Have all the patients been interviewed on the 30^th^ day of obtaining positive RT-PCR? If the duration between the test and interview was not uniform, please report the median duration. | Thank you for the very important comment. All the patients were interviewed on the 31^st^ day after RT-PCR positive. On the 30^th^ day, informed consent was taken from each patient and an appointment was taken for the next-day interview on the 31^st^ day. We have described details in the lines 181 – 185 in the ‘Materials and methods’ section. |
| 5. | Whereas some people with COVID-19 suffer severe, even life-threatening complications, others suffer no symptoms or just mild ones. How did the authors identify and manage this heterogeneity in the sample? | Thank you for the pertinent comment. Data were collected by telephone interview and it was not possible to differentiate the severity of the COVID-19 patients with verbal information as it will harvest recall bias. It could be considered as a limitation of this study which is mentioned in the lines 206-209 under the heading of ‘Data collection’ of the ‘Methods’ section and in the lines 514-517 of the ‘Discussion’ section. |
| 6. | Which all parameters / measures were used to ensure the quality, consistency, and relevancy of the data? | Thank you for the vital comment. Details of all measures are included in the lines 196-208 under the heading of ‘Data collection’ in the ‘Methods’ section. |
| 7. | Please provide the reference for grouping the response of question 1 of healthy days core module into ‘good health’ and ‘not so good health’. | Thank you for the crucial comment. Reference is added in the lines 218-220 under the heading of **‘Psychometric properties of the questionnaire’ in the ‘Material and methods; section**. |
| 8. | Line 175-177: Authors have reported that in addition to the response of question on general health (good health/ not so good health), the responses of nine additional items were treated as binary (yes/no). Shouldn’t it be eight additional items? | Thank you for the vital comment. It is corrected in the line 228 under the heading of **‘Psychometric properties of the questionnaire’ in ‘the Material and methods; section**. |
| 9. | Please provide details of the number of interviewers, their qualification, and their training process. Did authors investigate the variability among the responses obtained by different interviewers? | Thank you for the important comment. Details are given in the lines 178-181 and 202-208 under the heading of ‘Data collection’ in the ‘Materials and methods’ section. |
| 10. | Lines 189-201 may be shifted to discussion section. | Thank you for the crucial comment. The lines 189-201 describing the ‘advantages of CDC HRQOL-14 questionnaire’ are shifted to the discussion section. |
| 11. | In the method section, please write data on which sociodemographic characteristics were collected and being used in data analysis. | Thank you for the vital comment. Data, on which sociodemographic characteristics were collected and being used in data analysis are listed in the lines 189-191 under the heading of data collection in the ‘Methods’ section. |
| 12. | Is there any way to assess if the characteristics of the individuals excluded from the study were similar / different from those of included (lines 228-230)? | Thank you for the valuable comment. It is described in the 1^st^ paragraph of results section, total 1342 COVID-19 patients were identified who had completed 30 days after being diagnosed as COVID-19 positive by RT-PCR the during data collection period. The characteristics of the individuals excluded from the study were similar from those of included as the patients were from both sexes and all of them were aged >18 years. It was assessed by comparing their background characteristics obtained from the laboratory records. At the final stage, 1204 patients participated in the study, after excluding participants who had wrong contact number, didn`t attend the phone call, unwilling to participate and had an incomplete interview, from initially identified 1342 COVID-19 patients.. |
| 13. | The authors have described the advantages of using CDC HRQOL-14 (lines 189-201). However, there are other generic HRQOL measures which apart from providing health profiles of the patients, also provides the index-values (e.g., in range of -1 to +1), hence facilitate the calculation of QALYs and conduct of cost-utility analyses. Thereby, the authors are requested to provide the rationale of using CDC HRQOL-14, over the other HRQOL instruments. | Thank you for the vibrant comment. The rationale of using CDC HRQOL-14, over the other HRQOL instruments is given in the lines 113-123 of the ‘Introduction’ section and in the lines 519-539 of the ‘Discussion’ section. |
| 14. | Please discuss the validity of telephone administration of CDC HRQOL-14 by providing some relevant references. | Thank you for the pertinent comment. Reference (Reference 13) for the validity of telephone administration of CDC HRQOL-14 is provided. |
| 15. | Please mention if there was any respondent who answered ‘no’ while responding to question 1 of the activity limitation module? May provide this information in Table 2. | Thank you for the valuable comment. Question 1 of the activity limitation module (Duration of health related limited activity) was measured in days, which is a quantitative data. So, the respondents who answered ‘no’ to question 1 of the activity limitation module, their duration was recorded as ‘0’ days and accordingly, the mean value was calculated and provided in the Table 2 |
| 16. | Please comment in the results section if representativeness of the study population in comparison to the general population in Bangladesh was achieved. Which tests were used to assess the representativeness? The percentages of the larger population may be added to Table 1 to see the differences. | Thank you for the vial comment. The finding elated to the representativeness of the study population in comparison to the general population in Bangladesh is added in the lines 288-292 of the results section. The percentages of the larger population is added in texts of the Table-1. |
| 17. | Provide the information regarding overall unhealthy days. May add this in Table-2. | Thank you for the vital comment. Information regarding overall unhealthy days are added in Table-2. |
| 18. | Describe why the question 2 of the activity limitation module had been excluded? | Thank you for the crucial comment. Reasons for exclusion of question 2 of the activity limitation module are described in the lines 212-215 under ‘Psychometric properties of the questionnaire’ of the ‘Materials and methods’ section. |
| 19. | Please comment on the overlap of physically unhealthy days and mentally unhealthy days. Report the number and % of the respondents who had reported only physically unhealthy days, only mentally unhealthy days, and both (along with extent of overlap). | Thank you for the valuable comment. Table-2 is edited following the suggestions and related texts are rephrased accordingly in the lines 321-323 of the ‘Results’ section. |
| 20. | Provide details on the difference in the HRQOL among recovered and non-recovered COVID-19 patients (as mentioned in line118). | Thank you for the crucial comment. Difference between recovered and non-recovered COVID-19 patients is given in the lines 134-135 in ‘Material and methods’ section. |
| 21. | Please discuss why the mean duration of healthy days was higher among the patients having a symptom and comorbidities as compared to their counterparts. | Thank you for the very relevant comment. Actually, the mean duration of healthy days was lower among the patients having a symptom and comorbidities. So, information is corrected according to the results of the study in the lines 354–356 of the ‘Results’ section and discussed in the lines 487-489 of the ‘Discussion’ section. |
| 22. | A thorough copy-edit of the manuscript is required in terms of uniform use of abbreviations (using full-forms at the time of first use, and abbreviations at subsequent use), spelling and grammatical errors (e.g., lines 55, 56, 60, 94, 177, 232, 304-306, etc.), and table-footnotes (e.g., AOR in Table-3, use of single asterisk in Table-4). | Thank you for the very important comment. Copy-editing of the manuscript is done properly considering your valuable suggestions. |
| 23. | In figure-2, anosmia may be placed before shortness of breath. Likewise, in figure-3, chronic liver disease may be placed before chronic kidney disease. | Thank you for the specific comments. Based on the suggestions, figure 2 and figure 3 are edited. |

**Responses to reviewer-2 comments**

| **SL No** | **Comments** | **Responses** |
| --- | --- | --- |
| 1. | The rationale of the study needs to be well elaborated. | Thank you for the vital comment, Rationale of the study is now well elaborated. |
| 2. | Addition of studies using the same tool and specifically the used one will add more value. | Study on measuring the HRQOL of COVID-19 patients using the CDC HRQOL-14 questionnaire is very scarce. So, we are could not add more relevant studies using the same tool for comparison. |
| 3. | The methodology needs to be technically sounder. The sample size calculation reference needs to be incorporated. How eligibility criteria were developed? | Thank you for the vibrant comment. The ‘Methodology’ section is rewritten. Sorry, we could not add the reference for calculating sample size because sample size was based on the availability of all diagnosed COVID-19 patients on a selected month in a specific laboratory. The samples were selected purposively based on the specific selection criteria. For your kind attention, the selection criteria were developed based on the requirements of CDC HRQOL-14 questionnaire. |
| 4. | Results are quite confusing and need to be presented in a more understandable manner. The linear regression need to discussed in more detail with respect to different variables. | Thank you for the pertinent comment. The ‘Results’ section is rewritten in an understandable manner. |
| 5. | Discussion need to be rewritten. | Thank you for the vital comment. ‘Discussion’ section is rewritten and reorganized in a standard format. |
| 6. | Conclusion need to be rewritten with respect to study objectives. | Thank you for the crucial comment. Conclusion is rewritten with respect to the study objectives. |
| 7. | The quality of writing in terms of English language is poor. The article needs major editing. | Thank you for vital the comment. Comprehensive language editing of the article is done. |
