# Supplementary material for: Health-related quality of life of the adult COVID-19 patients following one-month illness experience since diagnosis: Findings of a cross-sectional study in Bangladesh
Source: PLoS One. 2023 Feb 22;18(2):e0279032. doi: 10.1371/journal.pone.0279032 (PMC9946204; doi:10.1371/journal.pone.0279032)
Supplement: Attachment — Submitted filename: Resonse to Reviewers.docx [file pone.0279032.s005.docx]

**Responses to comments of the reviewer 2**

**1. Comment:** While responding to my Comment-5 on the previous version (How did the authors identify and manage the heterogeneity in the COVID-19 severity among the sample?), the authors have mentioned that they have included this as a limitation in the lines 206-209 under the heading of ‘Data collection’ of the ‘Methods’ section and in the lines 514-517 of the ‘Discussion’ section. However, the lines at these places does not convey this meaning. I would like to request again that it should be mentioned in the manuscript in the explicit language, so that readers can draw the interpretation accordingly.

**Response:** Thank you for the crucial comment. The reasons for not being able to identify and manage the heterogeneity in the COVID-19 severity among the sample is mentioned in the manuscript in explicit language in the lines 442-446 of the ‘Discussion’ section.

**2. Comment:** While responding to my Comment-3 on the earlier version (how it was determined that a period of 30-days is appropriate for a COVID patient to get its HRQOL stabilized / returned to baseline), authors have described the appropriateness of the questionnaire to capture the HRQoL in the last 30 days. However, my concern remains unaddressed. I would highly appreciate if either it can be addressed, or explicitly mentioned as a limitation in the manuscript, so that the readers can interpret the results in a transparent manner.

**Response:** Thank you for your valuable comment. For your kind information, due to the lack of any previous baseline data, we could not determine whether the HRQoL of a COVID-19 patient stabilized or returned to baseline after 30 days. We only assessed the HRQoL of the COVID-19 patients for the period of one month since diagnosis (whether it was stabilized / returned to baseline or not) following the specific guidelines of the questionnaire, CDC HRQOL-14. This is added as a limitation of the study in the lines 451-457 of the ‘Discussion’ section. In addition, we compared the HRQOL by the background (not baseline) characteristics of the patients. Accordingly, corrections are made by using the term ‘background’ instead of ‘baseline’ characteristics (Tables 3, 4 and 5).

**3. Comment:** It will be very beneficial to the readers to know that how the authors investigated the variability among the responses obtained by different interviewers? The results of this analysis may be reported in a table.

**Response:** Thank you for the vibrant and valuable comment. For your kind information, we trained the interviewers extensively on telephone interviews and also conducted shadow interviews to minimize variability among the responses obtained by different interviewers which is mentioned in the lines 159-161 of the ‘Data Collection’ under the methods section.

**4. Comment:** Please explain the rationale of citing FAQs on CDC HRQOL-14 (Ref. no. 13) while describing the similarities between the sample and reference population (Lines 288-290)?

**Response:** Thank you for the crucial comment. For your kind consideration, that reference (Ref. no. 14) was cited mistakenly. The reference is corrected (Page 6) and no explanation for the rationale of citing FAQs is not required. Other references are also re-checked and accordingly those are rearranged throughout the article and in the reference list.

**5. Comment:** In my Comment-12 on the earlier version, I asked if the (clinical) characteristics of the individuals excluded from the study were similar / different from those of included? This information is usually reported to explain that the analysis has not omitted the patients of a particular severity, and the results can be interpreted accordingly.

**Response:** Thank you for your crucial comment. We collected data through telephone interviews and there was no scope to investigate and compare the clinical characteristics of the individuals excluded from the study were similar / different with those who were included. As a result, we it was not possible to be concerned about omitting the patients with particular severity. For example, some individuals were excluded who didn’t response to our telephone calls for three separate occasions and whose telephones were inactive. So, it was not possible to collect data on the severity form those excluded individuals and to compare it with the individuals who were included. It is mentioned in the lines 130-136 under the heading of study population in the ‘Materials and methods’ section.

**6. Comment:** While responding to my Comment-20 on the earlier version (Provide details on the difference in the HRQOL among recovered and non-recovered COVID-19 patients), the authors have mentioned that “Difference between recovered and non-recovered COVID-19 patients is given in the lines 134-135 in ‘Material and methods’ section. However, there is no mention of difference in HRQoL between recovered and non-recovered at this place. Secondly, please mention these differences in the ‘Results’ section, not in the ‘Methods’ section.

**Response:** Thank you for the valuable comment. For your kind information, we collected data on HRQOL from the COVID-19 patients who completed 30 days since diagnosis whether they were recovered or not-recovered. We had no data related to recovery because it was not possible to collect those data through telephone interviews without clinical evidences. So, we didn’t examine and compare the difference in the HRQOL between recovered and non-recovered COVID-19 patients separately. Moreover, this was not a primary concern of our study, hence, we didn’t conduct any analysis to find out the difference in the HRQO and mention in the results.

**7. Comment:** Please explain why the paper of Moriarty et al (The Center for Disease Control and Prevention's Healthy Days Measures–Population tracking of perceived physical and mental health over time) has been cited as different reference numbers at different places of the manuscript (first as 12, and then as 17)?

**Response:** Thank you for the dynamic comment. We are extremely sorry for this error. The reference no. 17 was a repetition of ref. no. 12. So , the ref. No 17 is deleted followed by, all the references are re-checked and rearranged throughout the article and in the reference list (Pages 24-25).
