# Supplementary material for: Health-related quality of life of the adult COVID-19 patients following one-month illness experience since diagnosis: Findings of a cross-sectional study in Bangladesh
Source: PLoS One. 2023 Feb 22;18(2):e0279032. doi: 10.1371/journal.pone.0279032 (PMC9946204; doi:10.1371/journal.pone.0279032)
Supplement: Attachment — Submitted filename: Reviewer Comments to Authors.docx [file pone.0279032.s003.docx]

This manuscript reported the HRQOL of COVID-19 patients in a cross-sectional sample in Bangladesh using CDC HRQOL-14 questionnaire. This is an important aspect of patient well-being, and the results should inform enhanced patient-specific support. Questions/ comments from my side are presented here:

1. Line 78: Please mention year along with the name of the month.
2. The data presented in lines 79-80 may be updated.
3. What data did authors use to inform that in COVID-19 patients, the duration of 30 days after the RT-PCR is appropriate to assess the effect of disease on their HRQOL (as their HRQOL is stabilised/ returned to baseline)? Is there a reference or any data to back up this determination?
4. Have all the patients been interviewed on the 30^th^ day of obtaining positive RT-PCR? If the duration between the test and interview was not uniform, please report the median duration.
5. Whereas some people with COVID-19 suffer severe, even life-threatening complications, others suffer no symptoms or just mild ones. How did the authors identify and manage this heterogeneity in the sample?
6. Which all parameters/ measures were used to ensure the quality, consistency, and relevancy of the data?
7. Please provide the reference for grouping the response of question 1 of healthy days core module into ‘good health’ and ‘not so good health’.
8. Line 175-177: Authors have reported that in addition to the response of question on general health (good health/ not so good health), the responses of nine additional items were treated as binary (yes/no). Shouldn’t it be eight additional items?
9. Please provide details of the number of interviewers, their qualification, and their training process. Did authors investigate the variability among the responses obtained by different interviewers?
10. Lines 189-201 may be shifted to discussion section.
11. In the method section, please write data on which sociodemographic characteristics were collected and being used in data analysis.
12. Is there any way to assess if the characteristics of the individuals excluded from the study were similar/ different from those of included (lines 228-230)?
13. The authors have described the advantages of using CDC HRQOL-14 (lines 189-201). However, there are other generic HRQOL measures which apart from providing health profiles of the patients, also provides the index-values (e.g., in range of -1 to +1), hence facilitate the calculation of QALYs and conduct of cost-utility analyses. Thereby, the authors are requested to provide the rationale of using CDC HRQOL-14, over the other HRQOL instruments.
14. Please discuss the validity of telephonic administration of CDC HRQOL-14 by providing some relevant references.
15. Please mention if there was any respondent who answered ‘no’ while responding to question 1 of the activity limitation module? May provide this information in Table 2.
16. Please comment in the results section if representativeness of the study population in comparison to the general population in Bangladesh was achieved. Which tests were used to assess the representativeness? The percentages of the larger population may be added to Table 1 to see the differences.
17. Provide the information regarding overall unhealthy days. May add this in Table-2.
18. Describe why the question 2 of the activity limitation module had been excluded?
19. Please comment on the overlap of physically unhealthy days and mentally unhealthy days. Report the number and % of the respondents who had reported only physically unhealthy days, only mentally unhealthy days, and both (along with extent of overlap).
20. Provide details on the difference in the HRQOL among recovered and non-recovered COVID-19 patients (as mentioned in line118).
21. Please discuss why the mean duration of healthy days was higher among the patients having a symptom and comorbidities as compared to their counterparts.
22. A thorough copy-edit of the manuscript is required in terms of uniform use of abbreviations (using full-forms at the time of first use, and abbreviations at subsequent use), spelling and grammatical errors (e.g., lines 55, 56, 60, 94, 177, 232, 304-306, etc.), and table-footnotes (e.g., AOR in Table-3, use of single asterisk in Table-4).
23. In figure-2, anosmia may be placed before shortness of breath. Likewise, in figure-3, chronic liver disease may be placed before chronic kidney disease.
